# ASCL1 promotes Scrt2 expression in the neural tube

**DOI:** 10.3389/fcell.2024.1324584

**Published:** 2024-04-05

**Authors:** Carolina Purcell Goes, Vitória Samartin Botezelli, Shirley Mirna De La Cruz, Mário Costa Cruz, Ana Paula Azambuja, Marcos Simoes-Costa, Chao Yun Irene Yan

**Affiliations:** ^1^ Department of Cell and Developmental Biology, Institute of Biomedical Sciences, University of São Paulo (USP), São Paulo, Brazil; ^2^ Facultad de Ciencias de la Salud, Universidad Científica del Sur, Lima, Peru; ^3^ Core Research Facilities (CEFAP), Institute of Biomedical Sciences, University of São Paulo (USP), São Paulo, Brazil; ^4^ Department of Molecular Biology and Genetics, Cornell University, Ithaca, NY, United States; ^5^ Department of Systems Biology, Boston Children’s Hospital, Harvard Medical School, Boston, MA, United States

**Keywords:** *Scratch2*, SCRT2, gene regulation, enhancer, ASCL1, POU3F2

## Abstract

ASCL1 is a transcription factor that directs neural progenitors towards lineage differentiation. Although many of the molecular mechanisms underlying its action have been described, several of its targets remain unidentified. We identified in the chick genome a putative enhancer (cE1) upstream of the transcription factor *Scratch2* (*Scrt2*) locus with a predicted heterodimerization motif for ASCL1 and POU3F2. In this study, we investigated the role of ASCL1 and this enhancer in regulating the expression of the *Scrt2* in the embryonic spinal cord. We confirmed that cE1 region interacted with the *Scrt2* promoter. cE1 was sufficient to mediate ASCL1-driven expression in the neural tube through the heterodimerization sites. Moreover, *Scrt2* expression was inhibited when we removed cE1 from the genome. These findings strongly indicate that ASCL1 regulates *Scrt2* transcription in the neural tube through cE1.

## Introduction

Neuronal differentiation is a complex process with multiple transition points. Key decisions include cell cycle arrest, commitment, and definition of neuronal lineage. In the developing spinal cord, cells are organized according to these decision points in concentric layers: the innermost ventricular zone harbors proliferating progenitors; after exiting the cell cycle these cells migrate externally to the intermediate zone. Within the intermediate zone, an intricate interplay of transcription factors along the dorsal-ventral axis establishes unique transcriptomic profiles, which ultimately determines spatial arrangement of different lineages, axonal projection, and neurochemical properties (Lai et al., 2016). The final steps of differentiation occur at the outermost layer, also known as the mantle zone (Angevine et al., 1970).

Changes in transcriptomic profiles is determined by a sequence of events that includes alterations in chromatin structure. Some of these alterations are initiated by pioneer transcription factors, which pave the way for the recruitment of chromatin remodeling complexes (Shlyueva et al., 2014). Beyond this, a second layer of control emerges through the interplay between transcription factors and genomic cis-regulatory regions. These regions exert their influence by modulating gene transcription upon motif recognition and binding. In summary, the dynamic orchestration of transcriptomic profiles hinges on chromatin structure, transcription factors and genomic cis-regulatory regions.

Transcription factors responsible for orchestrating early neural differentiation include proteins with bHLH domains (e.g., ASCL1, NEUROG2) or POU-homeodomains (e.g., POU3F2/BRN2). ASCL1 plays a pivotal role in neurogenesis by directly regulating the expression of multiple target genes, acting as a pioneer factor and binding to enhancers (Aydin et al., 2019). Recent ChIP-seq and RNA-seq assays have identified its target sequences in the mouse embryonic neural tube (Borromeo et al., 2014). The biological impact of ASCL1’s direct targets spans from controlling cell proliferation to various stages of neuronal differentiation and neurite outgrowth (Castro et al., 2011; Borromeo et al., 2014; Lin et al., 2018).

Despite considerable progress in understanding the role of ASCL1 on transcriptional control, some of its molecular mechanisms remain elusive, particularly the details of the interactions between ASCL1 and regulatory regions of its target genes. To shed light on this, we focused on a putative regulatory element near the coding region of *Scratch2* (*Scrt2*). *Scratch2* has been proposed to be a putative target for ASCL1 and POU3F2 (Castro et al., 2006). It encodes a zinc-finger transcription factor that is expressed in the neural tube, cranial and dorsal root ganglia. Its expression pattern and biological role in survival, differentiation, and migration are conserved across vertebrates (Marín and Nieto, 2006; Dam et al., 2011; Itoh et al., 2013). In the neural tube, its expression is restricted to a very tight domain, composed of early post-mitotic neural cells of the intermediate zone (Vieceli et al., 2013). The precise boundary of *Scrt2* expression domains suggests tight regulation of its expression and transcript availability (Goes et al., 2020).

Here, we present experimental data that confirms a direct interaction between the *Scrt2* promoter, and an enhancer controlled by ASCL1. ASCL1 regulates *Scrt2* expression through a ASCL1/POU3F2 heterodimerization motif. Together, these data validate previously reported *in silico* predictions and establishes a link between gene expression in the intermediate zone and ASCL1.

## Materials and methods

### *In silico* analysis

We used the BLAT tool in Genome Browser (GB) to find the heterodimerization motif (ATT[A/T]NCAT[A/T/G]CAG[C/G]TG) in the neighborhood of the *Scrt2* locus (Castro et al., 2006). We used the mouse genome (mm10) because it contains more epigenetic tracks available. E11.5 mouse neural tube epigenetic tracks were downloaded from GB for analysis using WashU Epigenome Browser (http://epigenomegateway.wustl.edu/browser/). We focused on the tracks for H3K27ac and H3K4me1 and susceptibility to DNAseI.

To identify the homologous sequence in chicken, we also used the Genome Browser platform. The mouse sequence was aligned with the chicken genome (galgal5) identifying a region located 2.6 kb upstream of *Scrt2* (cE1; chr20: 10,115,996–10,116,474). The GB PhyloP (Basewise Conservation by PhyloP) and MultiZ alignment parameters were applied to verify the mE1 and cE1 conservation amongst other vertebrates. To analyze the epigenetic profile of cE1, we generated the H3K27ac tracks by CUT&RUN experiments in neural tubes dissected from embryos in HH23 (see below).

We used the JASPAR 2022 platform to search for ASCL1 or POU3F2 binding sites (http://jaspar.genereg.net; Khan et al., 2018). We used thresholds of 70%. The mutant sequences of cE1 (M1 and M2) were generated by editing the motifs identified by JASPAR manually. The sequences (cE1, M1, and M2) are listed in the Supplementary Material. The Ep2 and Ep4 fragments were also submitted to JASPAR 2022 to search for ASCL1 and POU3F2 binding sites.

### CUT&RUN

Each experimental replicate was a pool of 5 HH23 neural tubes. The neural tubes were dissected and dissociated in Accumax (Innovative Cell Technologies, #AM105) for 30 min at room temperature under mild agitation. After dissociation, the assay was carried out as previously described (Skene and Henikoff, 2017). Briefly, cells were incubated with BioMag Plus Concanavalin A magnetic beads (Bangs Laboratories, BP531) and rabbit polyclonal anti-H3K27ac antibody (Abcam, cat. Ab177178) overnight at 4°C. Negative control was non-specific IgG and was processed in parallel with experimental samples (anti-IgG (Millipore, cat. #CS200621). After washing, protein A-Mnase was added to a final concentration of 700 ng/mL and incubated for 1 h at 4°C under agitation. The cells were washed and cooled to 0°C. 2 mM CaCl_2_ was added to activate Mnase for 45 min and terminated by the addition of 2XSTOP buffer (5M NaCl, 0.5M EDTA, 0.5M EGTA, 5% Digitonin, 2.5 µL RNAse A, 20 mg/mL Glycogen). The proteins were digested with proteinase K (10 mg/mL) for 10 min at 70°C and the DNA purified with phenol-chloroform and ethanol precipitation. The DNA was resuspended in water and quantified on Qubit (Invitrogen) with the HS Assay dsDNA kit (Invitrogen, cat. Q32851). Protein A–Mnase was kindly provided by Dr. Steven Henikoff (Howard Hughes Medical Institute, Seattle, EUA).

### CUT&RUN library construction and data analysis

The libraries were prepared with the NEBNext Ultra II DNA Library kit (NEB, cat. #E7645S). Equimolar concentrations of the libraries were pooled using the KAPA Library Quantification Kit (Roche, #07960336001) and sequenced on the NextSeq500 equipment (Illumina, United States).

Paired-end reads were trimmed using CutAdapt (Martin, 2011). Reads were filtered for those with a minimum length of 25bp or longer and aligned to the reference chicken galGal5 assembly using Bowtie2 (Langmead and Salzberg, 2012). Picard tool (GATK, Broad Institute) was used to mark duplicate reads and BAM files were filtered with SAMtools to discard unmapped reads, those which were not the primary alignment, reads failing platform/vendor quality checks, and PCR/optical duplicates (-f 2 -F 1804). Peak calling was performed using MACS2 (Zhang et al., 2008) with a q-value cutoff of 0.05 and normalized by the peaks for anti-IgG (negative control processed equally; Millipore, cat. #CS200621).

### Chromosome conformation capture (3C)-qPCR

The genomic regions that could interact with *Scrt2* were identified through H3K27ac peaks of the CUT&RUN assay in chick HH23 neural tubes. We searched for sequences near the *Scrt2* locus in HH23 neural tubes (grey outline of Figure 3). Each sample was a pool of 3 HH23 truncal neural tubes. The 3C assays followed previous protocols (Hagege et al., 2007; Naumova et al., 2012). In brief, the neural tubes were dissociated in Accumax and crosslinked in a final concentration of 1% formaldehyde. Then, the cells were washed in cold PBS with protease inhibitors and resuspended in lysis buffer for 10 min on ice. The lysate was resuspended in 1.2 × CutSmart (New England Biolabs) restriction buffer with 0.3% SDS and incubated in Thermomixer C (Eppendorf) at 37°C for 1 h at 900 rpm. Then, 2% Triton X-100 was added to the cell lysate and incubated at 37°C for 1 h at 900 rpm. An aliquot of 20 µL of undigested DNA was used as loading control. DNA was digested with 400U of NcoI (New England Biolabs, #R3193) at 37°C overnight. The enzyme was inactivated by adding 1.6% SDS and incubated at 65°C for 25 min. The lysates were incubated overnight at 16°C in T4 binding buffer (New England Biolabs, #M0202) with T4 DNA ligase (New England Biolabs, #M0202) for DNA intramolecular ligation. Then, the DNA was treated with 10 mg/mL Proteinase K overnight at 65°C. After treatment with 10 mg/mL RNAse, the DNA was purified with phenol-chloroform-Isoamyl alcohol (25:24:1), concentrated with Amicon Ultra-0.5 30K columns (Millipore, #UFC5030BK) and quantified on Qubit. The control sample was assembled by mixing equimolar amounts of eleven PCR products spanning the same 65 kb region of interest with minimal overlap. The primers for amplification of these products are listed in Supplementary Table S1. The amplification efficiency of primers pairs (fragment primers and constant primer) was verified through qPCR of DNA in a series of log titrations of control sample starting with 25 ng (Ea et al., 2015). The PCR was performed with Power SyBr Green (Applied Biosciences, cat. 4368577) at 60 °C on the ViiA 7 Real-Time PCR System (Applied Biosystems). Interaction frequencies were determined by the normalization of 3C experimental samples/loading control (GAPDH) as described previously (Splinter et al., 2003). The 3C-qPCR primers are listed in Supplementary Table S2. The 3C plot was constructed in R with the ggplot2 package.

### Cloning

The mouse *Pou3f2* sequence was acquired from Addgene (www.addgene.org; #19711) and subcloned by PCR into the pMES-IRES-GFP and pCI-IRES-H2B vectors. The rat *Ascl1* coding sequence in pCAGGS-GFP vector was provided by Dr. Diogo Castro (University of Porto, Portugal).

mE1 and cE1 were respectively PCR-amplified from mouse and chick genomic DNA using the primers described in the Supplementary Table S3 and cloned in the pTK-mRFP vector with HiFi DNA Assembly Master mix kit (NEB, cat. E2621). For luciferase assays, we subcloned the cE1 sequence into pGL3-Basic vector at KpnI and XhoI sites. For the CRISPR/Cas9, guides were designed adjacent to the PAM sequences found in cE1 and synthesized (IDT) with the sticky ends containing complementary sites for the restriction enzyme BsmBI (NEB, cat. R0580S). The oligonucleotides were hybridized and cloned into the pcU6.3-sgRNA vector (Williams et al., 2018). The scrambled oligonucleotides were generated with the “RNA sequence scrambler” tool (GenScript, United States, https://www.genscript.com/), using the chicken genome for off-target analysis. The sequence of each guide is described in Supplementary Table S4.

### Cell culture and luciferase assay

HEK293T cells were cultured in Dulbecco’s Modified Eagle’s Medium supplemented with 10% fetal bovine serum and 1% Penicillin/streptomycin, at 37°C and 5% CO_2_. For transfection, HEK293T cells were plated in 24-well plates 18–24 h before transfection, at a concentration of 3 × 10^5^ cells/well. Transfection was performed in triplicate and with simultaneous insertion of three different plasmids: one containing the gene coding for the tested transcription factor (*Ascl1* and/or *Pou3f2*), the pGL3-basic which encodes the firefly luciferase under cE1 control and pRL, the vector which produces *Renilla* luciferase that was used as a normalization factor. Empty pGL3-basic vector was used as control. Co-transfection was performed with 3.3 μL/well Polyethylenimine (PEI), 500 ng/well of transcription factor, 10 ng/well pRL, and 500 ng/well pGL3-cE1 overnight. Each sample was lysed in 1 × lysis buffer from Dual-Luciferase Reporter Assay System kit (Promega, cat. #E1910) and luciferase activity detection was performed according to manufacturer’s instructions in a Synergy HT luminometer (Biotek, United States).

### Chicken embryos

Fertilized eggs from *Gallus gallus* (Yamaguishi Farm, São Paulo, Brazil or University of Connecticut, Department of Animal Science–United States) were incubated at 37.8°C and 50% humidity until the desired developmental stages (Hamburger and Hamilton, 1992). All procedures were approved by institutional ethic committees (CEUA ICB/USP n° 025/2013) from University of São Paulo.

### In ovo electroporation

Single-sided electroporation of HH11-12 embryos followed standard protocols (Harada et al., 2017). Only the right halves of the neural tubes were electroporated in these assays. For *Pou3f2* or *Ascl1* overexpression, the plasmids were diluted in Fast Green 0.2% (Sigma, St. Louis, MI, United States of America) to the final concentration of 3 μg/μL. The plasmids containing mE1, cE1, USE, USE1, USE3, Ep2, Ep4 or empty pTKmRFP (3 μg/μL) were mixed in a proportion of 1:1 with the electroporation positive control pcDNA3.1-mGFP. For the CRISPR/Cas9 assays, we used pCAG-Cas9-2A:Citrine and pcU6.3-sgRNA. All the three vectors containing the cE1 sgRNAs (sgRNA1 to 3) or the scrambled (control) version of these guides, were co-electroporated with the vector containing Cas9. The electroporation parameters were: 5 pulses of 20V, 50 m of duration and 100 m of interval. Embryos were reincubated, screened for successful transfection, collected at stage HH23 and processed further accordingly.

For double-side electroporation, HH11-12 embryos were first electroporated in the left side with the control plasmids (pCDNA3.1-mGFP with pTK-cE1-mRFP or pTK-M1-mRFP or pTK-M2-mRFP). After 3 h, the contralateral (right) side was electroporated with a plasmid mix containing either of the assay plasmids and the plasmids coding for the transcription factors ASCL1 or POU3F2 and GFP (*Ascl1* with pTK-cE1-mRFP or pTK-M1-mRFP or pTK-M2-mRFP; *Pou3f2* with pTK-cE1-mRFP or pTK-M1-mRFP or pTK-M2-mRFP) using the same parameters described previously but with inverted electrode polarity so the plasmid would migrate to the other hemitube. After electroporation, the embryos were reincubated and collected at HH17-18. The embryos were fixed in 4% paraformaldehyde (PFA) washed in 1x PBS and photographed using the Axiozoom V16 fluorescence stereoscope.

Dorsal images of each embryo were measured for pixel intensity across the mediolateral axis. The GFP and mRFP intensity were determined with the “Profile” tool of ZEN Blue v2.6 software (Zeiss) (Supplementary Figure S7). The curves were plotted and the area under the curve (AUC) was obtained for the left and right sides in the GraphPad Prism v.9 software. The magenta line on the graph refers to pixel values generated in the red channel (mRFP) and the cyan to green channel (mGFP) (Figure 5A). The electroporation effectiveness was considered for each hemitube through the mRFP/mGFP ratio.

### *In situ* hybridization (ISH) and immunohistochemistry

Whole mount ISH was performed on stage HH22-23 embryos after electroporation following standard procedures (Acloque et al., 2008). Thereafter, the embryos were imaged with Nikon SMZ1500 stereomicroscope and then embedded in gelatin-sucrose medium for cryosectioning (Bronner-Fraser et al., 1996). Cross sections with 25 μm of trunk neural tube were collected in SuperFrost slides (Fisher Scientifics, Waltham, MA, United States). When there was a need for immunohistochemistry after *in situ* hybridization, we used rabbit anti-GFP IgG (1:400, Molecular Probes, cat. A-6455) and anti-FLAG (1:500, Sigma Aldrich, cat. F3165–mouse M2) primary antibodies. The secondary antibodies were goat anti-rabbit IgG coupled to Alexa 488 (dilution 1: 200, Molecular Probes, cat. A-11008) and goat anti-mouse igG-Alexa 568 (dilution 1:500, Molecular Probes, cat. A11004). Finally, the slides were washed in PBS three times, mounted in FluoroShield Mounting (with DAPI, Abcam, Cambridge, Cambridgeshire, United Kingdom) and viewed under a fluorescence microscope (Zeiss Axio Imager. D2 with Axiocam 503 Color camera or Zeiss Imager. Z2 with Zeiss Axiocam 506 mono camera) or ZeisscAxioVert.A1 microscope with attached Zeiss AxioCam Icm1 camera (CEFAP, USP).

For ISH on cryosections, HH23 embryos were cryosectioned at 12 μm. The slides were used immediately after sectioning and dried at 37°C for 2 h. The riboprobe was added to the slides in Hyb solution (50% Formamide, 10% 10X Salt Solution, 5 g dextran sulfate, 50 mg yeast RNA and 1% 100X Denhardt’s solution in DEPC-treated water) and incubated overnight at 68°C for *Ascl1*, *Pou3f2* and *Scrt2*. Then, the slides were washed and incubated with a blocking solution (10% NGS in 1x TBS + 0.5% Tween-20) for 1 h at room temperature followed by incubation overnight at 4°C with anti-DIG (1:2000, Roche) in blocking solution (1% NGS in 1x TBS + 0.5% Tween-20). On the third day, slides were washed and the labelling was detected with NBT/BCIP (Roche) diluted at 2 μL/mL in NTMT (2% 5M NaCl, 10% 1M Tris pH9.5, 2.5% 2M MgCl2, 1% Tween-20 10% in water) at room temperature until the staining appeared. Finally, slides were post-fixed in 4% PFA for 30 min, washed with 1x PBS and mounted with Fluoromount-g (Invitrogen, 00-4958-02). Images were captured with ZEISS Axiocam 503 Color camera coupled with ZEISS Axio Imager D2 microscope.

### Immunohistochemistry

Fixed embryos were cryoprotected in 20% sucrose overnight at 4°C. Then, the embryos were embedded in 20% sucrose:TissueTek OCT (Sakura, Alphen aan den Rijn, South Holland, Netherlands), sectioned in a cryostat (Leica, CM1850 UV), and collected on gelatin-coated slides. The cryosections were dried, washed with PBS and blocked with 3% NGS (Normal Goat Serum, Jackson Immunoresearch, West Grove, PA, United States) in PBST (PBS with Triton X-100 0.2%, Sigma) for 1h at room temperature in a humid chamber. The primary antibody was diluted in blocking solution and incubated overnight in a humid chamber at room temperature. The primary antibodies used were anti-DsRed2 mouse (1:50; Santa Cruz Biotechnology, cat. Sc-101526), rabbit anti-GFP IgG (1:200; Molecular Probes, cat. A-6455) and mouse anti-HNK-1 IgM (DSHB, cat. 3H5). The secondary antibodies were incubated for 2 h at room temperature and in a humid chamber. The secondary antibodies used were goat anti-mouse IgM coupled to Alexa 488 (1:200, Molecular Probes, cat. A-21042), goat anti-rabbit IgG -Alexa 488 (1:200, Molecular Probes, cat. A-11008), donkey anti-rabbit IgG - Alexa 647 (1:800, Molecular Probes, cat. A31573), goat anti-rabbit IgG–Alexa 568 (1:500, Molecular Probes, cat. A11011) and goat anti-mouse IgG–Alexa 568 (1:500, Molecular Probes, cat. A11004). Finally, the slides were washed and mounted in FluoroShield with DAPI.

### Quantification of expression fields

We developed a method based on previous works (Palmesino et al., 2010; Kabayiza et al., 2017; Gray de Cristoforis et al., 2020) to calculate the cell position and distance from the center of the neural tube using *in situ* hybridization images. Our approach involved a two-step process: cell identification using deep learning and subsequent data processing with a Python script (Supplementary Figure S1A). To achieve this, we trained a model with a few representative ISH images that were manually annotated to identify individual cells in Cellpose software (Pachitariu and Stringer, 2022). Once the model was trained, we applied it to the CellProfiler software with the integrated RunCellpose plugin (Stirling et al., 2021; Weisbart et al., 2023). This combination allows us to create a pipeline to perform automated and unbiased identification and segmentation of the neural tube sides, cell segmentation inside the neural tube, and determination of cell coordinates (X and Y) relative to a central line that bisects the neural tube. Subsequently, the extracted data underwent processing using a Python script implemented on Colab. The script was designed to calculate the distances of cells from the center of the neural tube. The normalization step was the perpendicular distance of each cell to the neural tube midline over the Euclidean distance of the two points that defines the neural tube midline. It involved post-processing steps for distance computation and the generation of graphical representations.

A more detailed understanding of our methodology and the pipeline used can be found on our GitHub repository:

https://github.com/mccruz07/Goes_CP_ASCL1_promotes_SCRT2_expression_in_the_neural_tube.

### Statistical analysis

Statistical analyses were performed with Graphpad Prism v.9 software by first checking data normality and then by applying the specific test for each data. This information can be found in the figure legends. To compare changes on distribution of individual cell coordinates (X and Y values) in 2D density data from experimental and control samples, we applied the Two-sample Hotelling’s T^2^ test using the R package Hotelling (v. 1.0–8) (Palmesino et al., 2010).

## Results

### ASCL1 *and* POU3F2 *modulate Scrt2 expression in the embryo*

**FIGURE 1:**
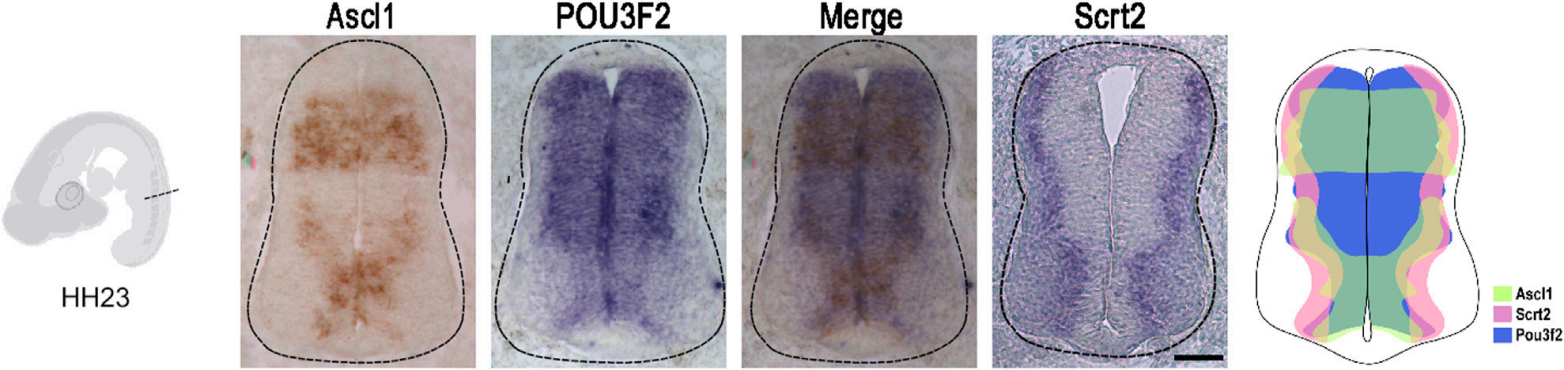
*Ascl1* and *Pou3f2* are co-expressed with *Scrt2* in the chick neural tube. *In situ* hybridization of *Ascl1, Pou3f2* and *Scrt2* in HH23 chicken neural tubes. The diagram represents the expression overlap of these genes. Scale bar: 50 µm.

**FIGURE 2:**
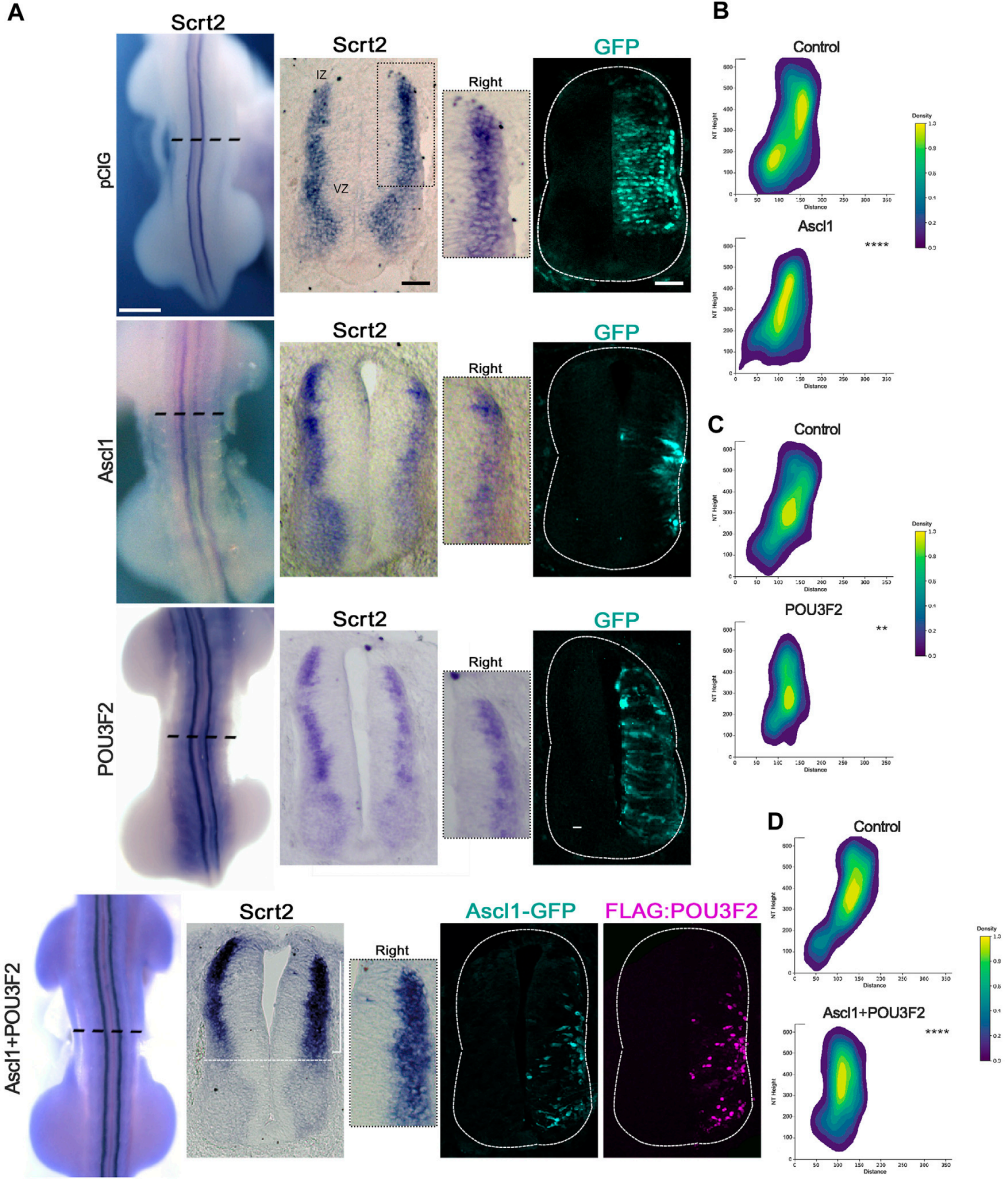
ASCL1 and POU3F2 modulate *Scrt2* expression. **(A)** HH23 Whole mount embryos electroporated at the right hemitube with pCIG (control plasmid) (*n* = 3), *Ascl1* (*n* = 6) *Pou3f2* (*n* = 4) or *Ascl1+Pou3f2* (*n* = 14). The bracket indicates the increase in *Scrt2* expression post overexpression of *Ascl1+Pou3f2*. Immunolabeling for GFP and FLAG was used to assess the electroporation area of each condition. The insets show the magnification of the dorsal domain of each hemitube. **(B–D)** 2D density plots of *Scrt2*-positive cells in each hemitube. **(B)** *Ascl1* (*n* = 11 sections from 6 embryos), **(C)** *Pou3f2* (*n* = 18 sections from 5 embryos) and **(D)** *Ascl1+Pou3f2* (*n* = 20 sections from 10 embryos) (Two-sample Hotelling’s T^2^ test, ** *p* < 0.01; **** *p* < 0.0001). Whole mount and sections scale bars are 250 µm and 50 μm, respectively.

If *Scrt2* expression is modulated by ASCL1 and POU3F2, it is expected that some *Scrt2*-positive cells would co-express *Ascl1* and/or *Pou3f2*. In other words, *Scrt2* expression pattern should overlap with that of *Ascl1/Pou3f2*. Indeed, the dorsal region of the intermediate zone contains *Scrt2/Pou3f2/Ascl1* positive cells with a significant overlap (Figure 1). To verify whether ASCL1 and/or POU3F2 modulate *Scrt2* expression, we overexpressed them individually and jointly. Overexpression of *Ascl1* or *Pou3f2* alone reduced *Scrt2* expression field when compared to the contralateral side and with the control embryo (Figures 2A–C). However, when *Ascl1* and *Pou3f2* were overexpressed jointly, *Scrt2* expression field increased in the dorsal domain of the neural tube (Figures 2A–D). We quantified these changes with 2D density plots of the distribution of *Scrt2*-positive cells in each section and the above-mentioned changes were statistically significant (Figures 2B–D; Supplementary Figure S1B).

**FIGURE 3:**
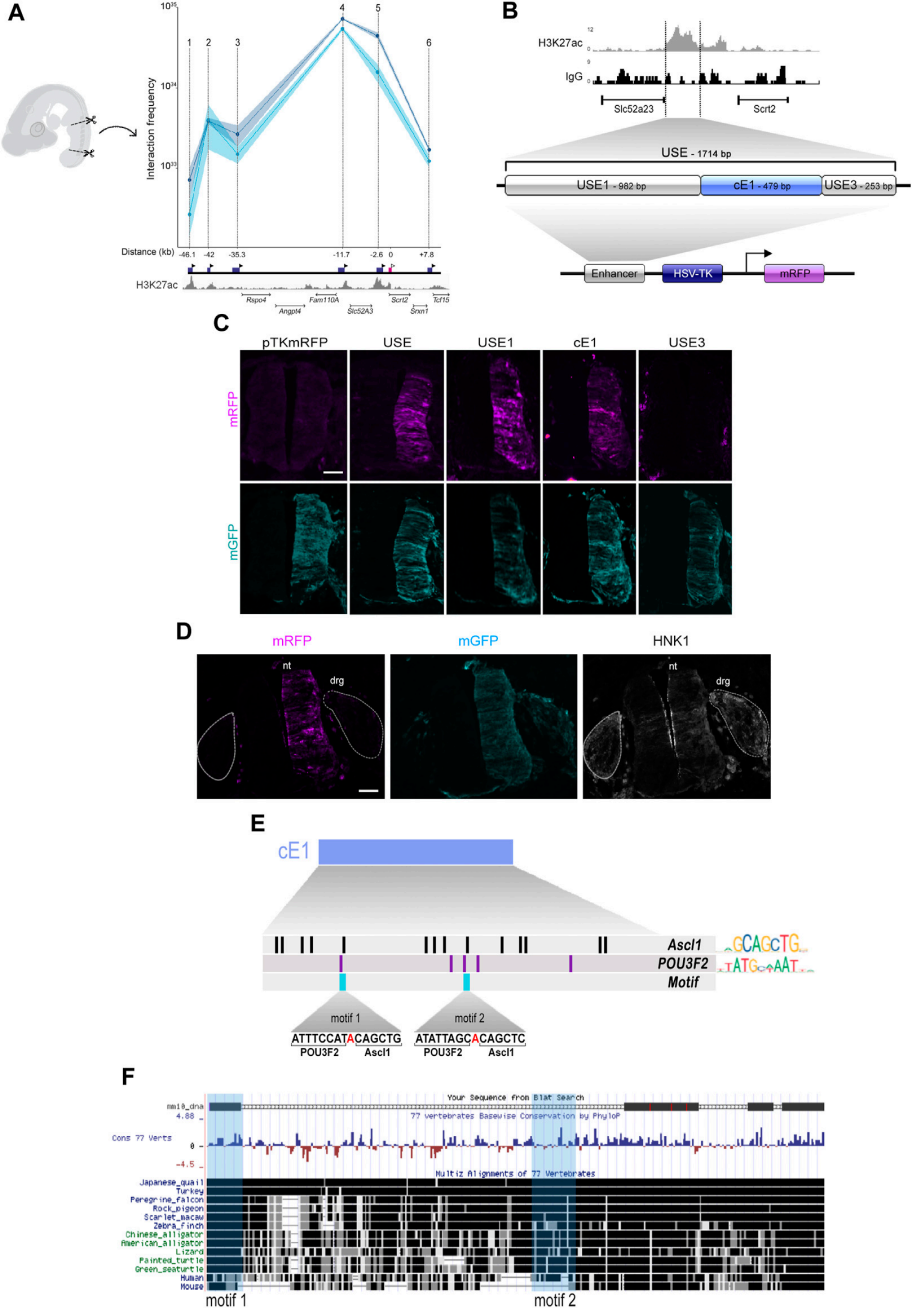
The cE1 genomic region has high levels of H3K27ac in the neural tube and interacts with *Scrt2* promoter. **(A)** 3C-qPCR assay (*n* = 2) shows the interaction of specific genomic regions (indicated by points 1-6) with the *Scrt2* promoter. The dark blue boxes indicate the relative genomic position of the regions, and the black arrowheads indicates the position of each primer used in the qPCR for detection of interactions. The pink box marks the *Scrt2* promoter region, and the white arrowhead indicates the location of its constant primer. The numbers below the dark blue boxes indicate the distance (kb) of each candidate enhancer relative to *Scrt2* promoter (pink box). Dark and light blue curves represent the biological replicates of the 3C experiment. The shaded error bands represent ±s.e.m. **(B)** Detailed view of the epigenomic landscape of H3K27ac mark in peak 5 and its neighboring regions. We renamed peak 5 as USE. Nonspecific IgG was used as control. USE was subdivided into USE1, cE1 and USE3 and cloned in pTK-mRFP vector. **(C)** Full length USE and its subregions were tested for their ability to drive mRFP expression in the HH23 neural tube. Empty pTK-mRFP vector was used as negative control and pCDNA3.1-mGFP was coeletroporated in all samples as a tracer for electroporation effectiveness. USE (*n* = 12 embryos), USE1 (*n* = 6 embryos), cE1 (*n* = 9 embryos) or USE3 (*n* = 5 embryos). Scale bar: 50 µm. **(D)** cE1-mRFP and pCDNA3.1-mGFP were coelectroporated in the dorsal root ganglion (drg). Immunolabeling for mRFP (magenta), GFP (cyan), and HNK1 (grey) show that mRFP is absent in drg. nt = neural tube. **(E)** A detailed view of cE1, highlighting the potential binding sites for ASCL1 and POU3F2. Each vertical bar indicates the position of a potential binding site for ASCL1 (black) and POU3F2 (purple). The light blue bars show the heterodimerization motifs. **(F)** mE1 aligns to the chicken genome (galGal6) at its homologue cE1. Fragments of mE1 are conserved among 77 vertebrates. The light blue vertical boxes indicate the position of the POU3F2/ASCL1 heterodimerization motifs.

To verify if ASCL1 or POU3F2 could modulate *Scrt2* transcription directly, we first searched for potential regulatory elements in the genomic region surrounding *Scrt2* locus. For this, we used CUT&RUN tracks from the epigenetic indicator of active element Histone 3 acetylated at lysine 27 (H3K27ac) in HH23 chick neural tube. Briefly, the principle of this assay is similar to that of ChIP-seq in that it identifies genomic regions enriched for a specific target. We identified six H3K27ac-enriched regions near the *Scrt2* locus as potential enhancers. Next, we verified if any of these regions could interact with *Scrt2* promoter to modulate its transcription through a chromatin conformation assay (3C) (Figure 3A). Points on the graph correspond to the relative frequency of the interaction between the promoter and each individual candidate enhancer (Figure 3A). The highest peaks, i.e., had higher frequency of interaction with *Scrt2* promoter, were peak 5, peaks 4 (Ep4) and 2 (Ep2). Ep2 and Ep4 are putative new *Scrt2* promoter-interacting regions located 42 kb and 11.7 kb upstream from the *Scrt2* promoter, respectively. Ep5 was previously identified in mouse as a candidate regulatory element targeting *Scrt2* (Castro et al., 2006).

Since *Scrt2* is expressed in other neural tissues besides the neural tube (Vieceli et al., 2013), our next step was to analyze if the genomic elements corresponding to peaks 2, 4 and 5 could modulate gene expression in this tissue (Figure 3B). Both peak 2 and 5 promoted mRFP expression in the neural tube (Figures 3C; Supplementary Figure S2B) whereas peak 4 did not (Supplementary Figure S4E). However, we did not find regions homologous to peak 2 in the genome of other species, suggesting that it was a feature of birds genome. Thus, we focused our efforts on peak 5, which we renamed as USE (*Upstream Enhancer*). We then searched for a core region that is more active by subdividing USE into three fragments, that were tested separately: USE1 (982bp), cE1 (479bp) and USE3 (253bp) (Figure 3B).

Subregions USE1 and cE1 directed the expression of mRFP and generated an expression pattern like that of the complete USE sequence (Figure 3C). USE3 did not promote expression of mRFP (Figure 3C), suggesting that it has no enhancer properties by itself. *Scrt2* expression in the posterior neural tube starts at stage HH15 and remains until at least HH30 (Vieceli et al., 2013). To verify whether cE1 could drive gene expression during the entire period in which endogenous *Scrt2* is present, we analysed cE1 in the same time course (Supplementary Figure S3). Indeed, cE1 can drive the expression of mRFP in embryos from stage HH15 through HH23.

Although the dorsal root ganglion expresses *Scrt2* (Vieceli et al., 2013), neither USE or any of its subregions promoted mRFP expression in the ganglia (Figure 3D). Thus, the enhancer activity remained restricted to the neural tube. Together, these data suggest that cE1 is an enhancer element that drives *Scrt2* expression specifically in the neural tube.

Since *Scrt2* expression pattern is conserved amongst vertebrates, we looked for a homologous region in the mouse genome. We named the mouse region homologous to cE1 as mE1. It is located 13 kb upstream of *Scrt2* (mm9 chr2: 151,894,401-151,896,000; mm10 chr2:152,068,658-152,070,265). mE1 contains a previously reported ASCL1/POU3F2 heterodimerization site (Castro et al., 2006). Moreover, the ENCODE enhancer mapping analysis showed that this region presents epigenetic signatures characteristic of transcriptionally active sequences in E11.5 mouse neural tube. Specifically, it shows a high incidence of the active enhancer markers H3K27ac, H3K4me1 and chromatin availability (mm10; Supplementary Figure S4A). We cloned mE1 and tested its ability to drive mGFP expression in the neural tube. mE1 enhancer activity was similar to cE1 and remained active from HH15 until at least HH23 (Supplementary Figure S4B). This supports the idea that E1 is an evolutionary conserved enhancer active in embryonic neural tube.

We also searched for potential binding sites for ASCL1 and POU3F2 in cE1 and found 14 sites for ASCL1 and five sites for POU3F2. Of these, two ASCL1 and POU3F2 sites were adjoining, like the heterodimerization motif identified in mE1 (Castro et al., 2006), and were named motifs 1 and 2 (Figure 3E). The heterodimerization motif was previously proposed as a sequence that promotes interaction of ASCL1/POU3F2, resulting in synergistic activation of transcription of *Delta* (Castro et al., 2006). In cE1, motif 1 is entirely conserved in multiple vertebrates, including mouse and human, whereas the motif 2 is present in chicken and human but not in mouse (Figure 3F).

### cE1 modulates Scrt2 expression

**FIGURE 4:**
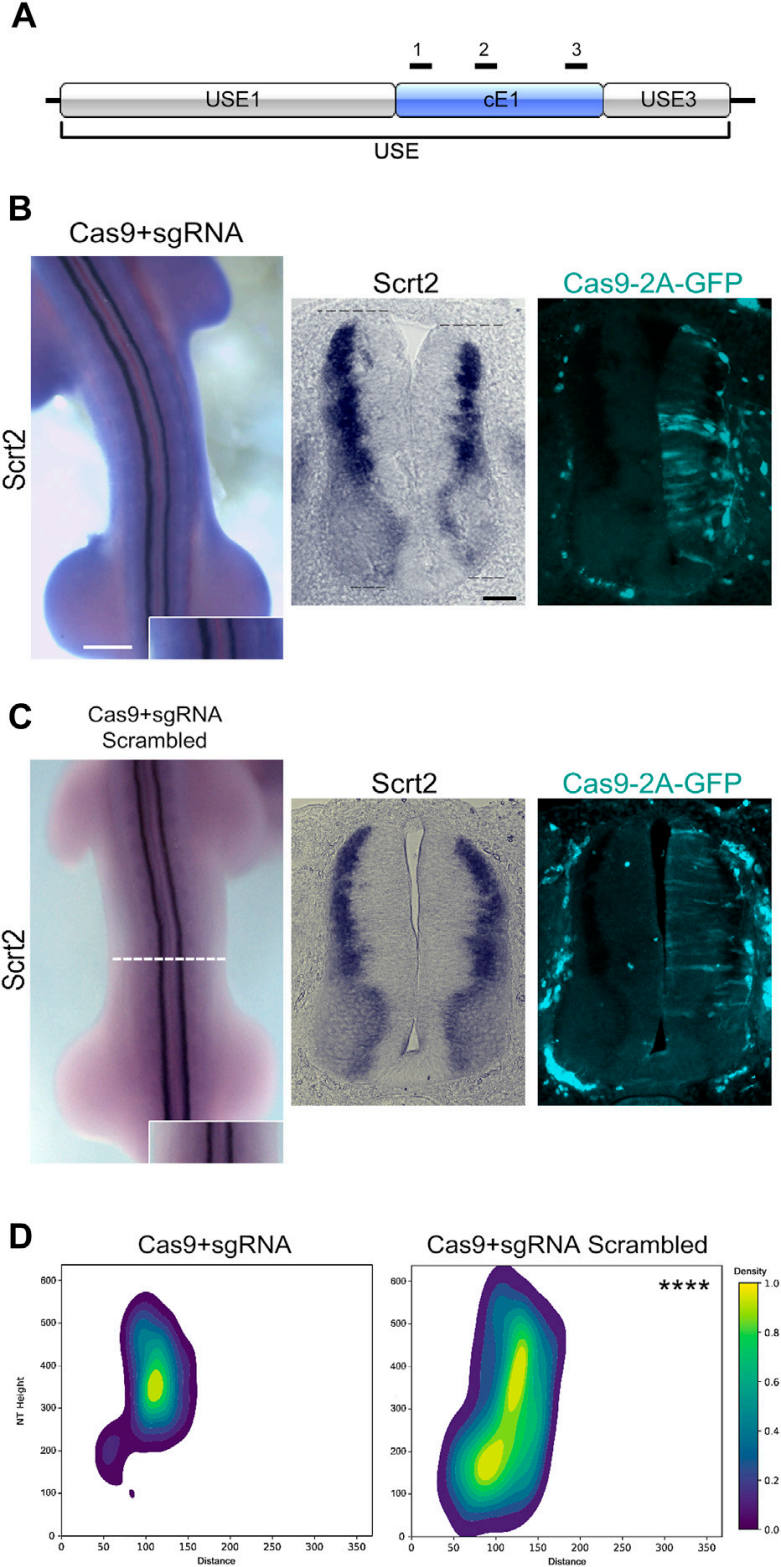
Genomic removal of cE1 reduces *Scrt2* expression field. **(A)** The sgRNAs (black bars) used in all CRISPR/Cas9-mediated genomic and epigenomic editing were targeted to multiple sites in cE1. All electroporations were on the right hemitube, and the electroporated cells can be identified by the presence of GFP. **(B)** Whole mount HH23 embryos electroporated with CRISPR/Cas9 and the combination of all sgRNAs show reduced *Scrt2* expression field in width, and there is a slight shortening of the dorsoventral axis of the neural tube (dashed lines; *n* = 14). **(C)** Control embryos electroporated with CRISPR/Cas9 and scrambled sgRNAs (*n* = 5). **(D)** 2D density plots of *Scrt2*-positive cells in the right hemitube of sgRNA (*n* = 20 sections from 14 embryos) and sgRNA scrambled (*n* = 6 sections from 5 embryos). (Two-sample Hotelling’s T^2^ test, **** *p* < 0.0001). Whole mount and sections scale bars are 250 µm and 50 μm, respectively.

To verify if cE1 modulates *Scrt2* expression, we removed cE1 from the genome with CRISPR/Cas9 (Figure 4A). *Scrt2* expression domain was significantly reduced in the CRISPR-edited side when compared to the contralateral control side that was not electroporated (Figures 4B, D). Moreover, the electroporated side of the neural tube was shorter in the dorsoventral axis. In contrast, embryos electroporated with scrambled sgRNA did not show macro or microscopic changes (Figures 4C, D). Quantification of the expression fields after their conversion to 2D density plots confirmed the significant reduction of *Scrt2* expression domain post genomic removal of cE1 when compared to the embryos electroporated with scrambled sgRNAs (Figure 4D; Supplementary Figure S5).

### ASCL1 *promotes transcription through cE1*

**FIGURE 5:**
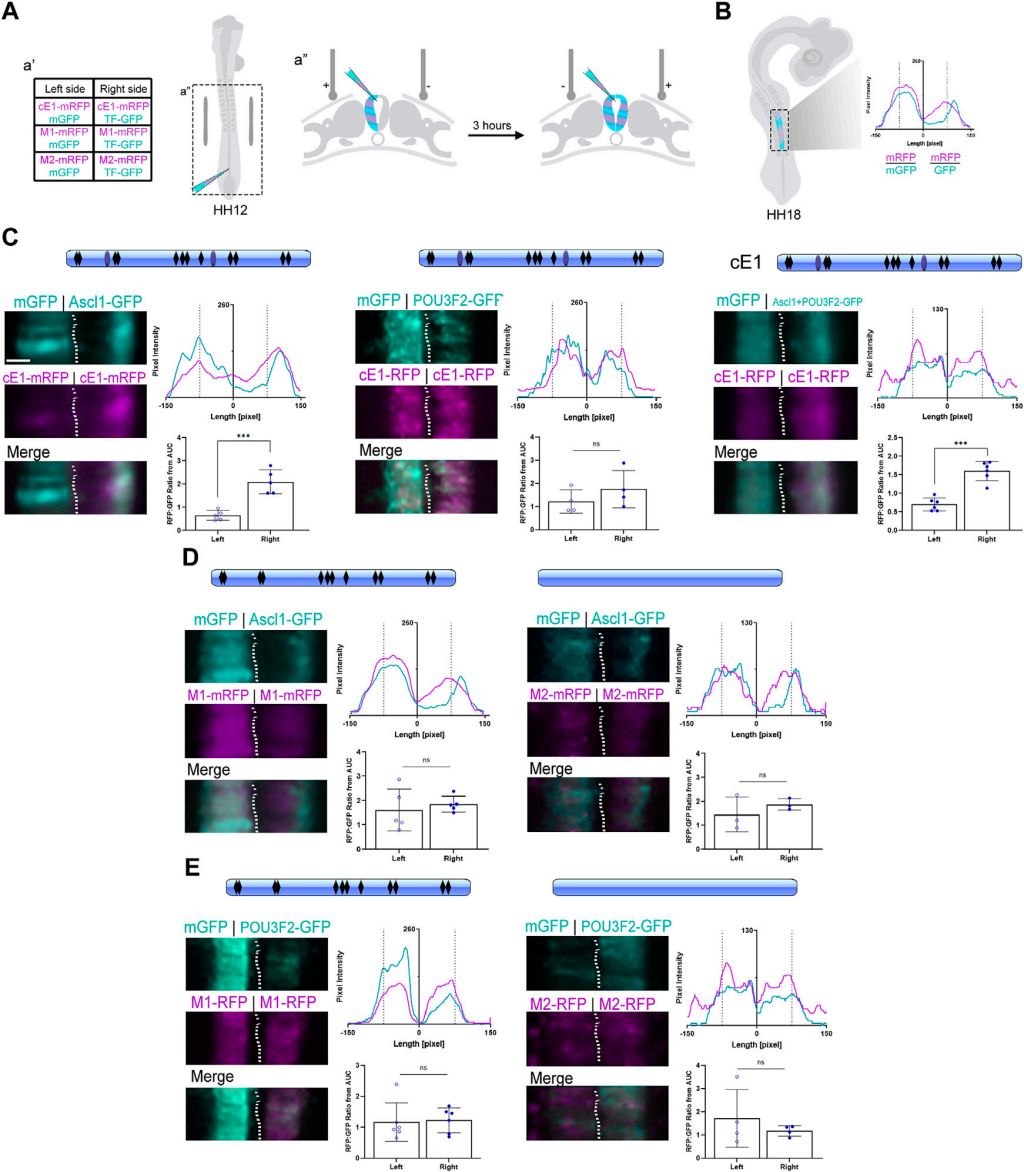
ASCL1 modulates transcription in the neural tube through heterodimerization sites in cE1. **(A)** Graphical representation of the bilateral electroporation procedure (details in Methods). **(B)** Graphical representation of the fluorescence levels quantification of electroporated neural tubes at HH18 (details in Methods). **(C)** cE1 contained the predicted binding sites for ASCL1 (black rhomboids) as well as the heterodimerization sites (light blue ovals). *Ascl1*, but not *Pou3f2*, increased mRFP transcription from cE1-mRFP (*n* = 5 and 4, respectively; Paired Student’s t-test, *** *p* < 0.001). In contrast, co-electroporation of *Ascl1+Pou3f2* increased mRFP expression through cE1 (*n* = 6; Paired Student’s *t*-test, *** *p* < 0.001). **(D)** The M1 construct was mutated for the POU3F2/ASCL1 heterodimerization motifs (no more blue ovals) and in the M2 construct all predicted ASCL1 binding sites were removed (no blue ovals or black rhomboids). Embryos were coelectroporated with **(D)** M1+*Ascl1* (*n* = 5), M2+*Ascl1* (*n* = 3) or **(E)** *Pou3f2*+M1 (*n* = 6) or *Pou3f2*+M2 (*n* = 4). No difference was observed in mRFP expression relative to the control right hemitube in any condition; the expression of mRFP remained at basal levels in both hemitubes for all conditions. Scale bar: 65 µm.

Finally, we asked if ASCL1 and POU3F2 could modulate *Scrt2* expression by interacting directly with cE1. We first tested if ASCL1 or POU3F2 interact with cE1 *in vitro* using luciferase assays in HEK293T cells (Supplementary Figure S6). Thereafter, we used an *in-embryo* transcription assay based on mRFP expression levels. Briefly, cE1-mRFP was coelectroporated with mGFP in the left half of HH12 embryos’ neural tube (Figure 5A). Subsequently, the contralateral hemitube was electroporated with cE1-mFRP and *Ascl1*-GFP/*Pou3f2*-GFP alone or jointly. In this setup, mRFP expression indicates transcriptional activity. To compensate for differences in electroporation efficiency between different embryos and hemitubes, we generated pixel intensity line plots, determined the area under the curve values and normalized the mRFP signal with that of GFP (Figure 5B; Supplementary Figure S7). The GFP signal in the *Ascl1*-eletroporated side was higher in the external layer of the neural tube, confirming that ASCL1 was active and promoting the premature migratory behaviour reported previously (Figure 5C; Castro et al., 2006).

When *Ascl1* was overexpressed, the peak signal in the mRFP channel overlapped with the peak signal in the GFP channel, suggesting a cell autonomous effect of ASCL1 in promoting cE1-mediated mRFP transcription (Figure 5C). In contrast, overexpression of *Pou3f2* did not change the mRFP/GFP ratio in comparison to the control contralateral hemitube (Figure 5C). Joint overexpression of *Ascl1* and *Pou3f2* also promoted mRFP transcription (Figure 5C). Together, these results suggest that ASCL1 but not POU3F2 drove cE1-mediated mRFP expression.

Our next step was to remove the specific motifs in cE1 that could be recognized by ASCL1 and POU3F2. Thus, we generated two mutated versions of cE1: M1 lacks the two predicted ASCL1/POU3F2 heterodimerization motifs (Figure 3E). M2 lacks all the ASCL1 predicted target sites - including the heterodimerization motifs. When *Ascl1* was coexpressed with either of these mutants, the mRFP/GFP ratio remained at basal levels, equal to the control conditions (Figure 5D). POU3F2 did not modulate transcription either with M1 or M2 constructs (Figure 5E). Together, these data suggest that ASCL1 modulates cE1-driven transcription through the ASCL1/POU3F2 heterodimerization motifs.

## Discussion

The genes in the intermediate zone of the neural tube are expressed in a very narrow strip, sandwiched between the domains of neurogenesis and differentiation. This pattern suggests that they could be direct targets for neurogenic transcription factors present in more internal layers. Here, we investigated the mechanism whereby ASCL1 regulates gene expression in this region by focusing on *Scrt2* as a putative target gene. We identified an evolutionarily conserved enhancer element near the transcription start site of chick *Scrt2* that harbors predicted heterodimerization sites for ASCL1/POU3F2.

The mouse homologue of this enhancer was previously reported in a genome-wide search for regions that contained this ASCL1/POU3F2-binding motif (Castro et al., 2006). The sequence of the motif was defined through a series of mutagenesis experiments followed by gel shift and luciferase reporter assay. The motif promotes heterodimerization of ASCL1/POU3F2, resulting in their synergistic effect on the expression of *Delta*. This motif was detected in several regions close to the transcription start site of neural genes–including *Scrt2*. Indeed, ChIP-seq assays confirmed that ASCL1 binds to the same motif near *Scrt2* that we identified here (Woods et al., 2022). However, the relationship between this candidate enhancer and *Scrt2* as its corresponding target gene, was not shown. Here, we confirmed that cE1 interacts with the *Scrt2* promoter. Additionally, deletion of cE1 reduced *Scrt2* expression.

cE1 was sufficient to promote ASCL1-driven neural expression in the embryo. Furthermore, the ASCL1-driven gene expression occurred through the predicted heterodimerization motifs. Their removal abolished ASCL1-driven gene reporter transcription. In contrast, POU3F2 by itself could not drive transcription through cE1. The simplest possibility is that POU3F2 does not interact with cE1. When added together with ASCL1, it did not synergize or compete with ASCL1 for transcriptional control.

An alternative explanation is that ASCL1 must first bind to cE1 before recruiting POU3F2. This is supported by the ASCL1 role as a pioneer factor, which binds to closed chromatin and promote the chromatin accessibility (Raposo et al., 2015). Also, in certain occasions, POU3F2 itself is insufficient to induce expression. For instance, POU3F2 binds to *Pax3* promoter as a monomer but requires co-expression of *Hoxa1* to activate transcription (Pruitt et al., 2004). Likewise, POU3F2 alone does not activate an enhancer for *Delta*, but recruits and synergizes with ASCL1 (Castro et al., 2006). If this scenario is applied to explain our results, ASCL1 must be present prior to POU3F2 overexpression to facilitate the interaction of POU3F2 with cE1 and promote transcription. However, as seen in the *in situ* hybridization data, the innermost limit of *Ascl1* domain is more external to the innermost expression domain of *Pou3f2*. Considering the internal-external gradient of cell maturity organization in the neural tube, this suggests that *Ascl1* is expressed after *Pou3f2*. Therefore, overexpression of *Pou3f2* alone would be insufficient to promote transcription because endogenous *Ascl1* is not yet present.

The effect of *Ascl1* and *Pou3f2*, overexpressed singly, on *Scrt2* expression domain differed from the cE1-promoted neural tube transcriptional assays. An increase in either ASCL1 or POU3F2 reduced the expression of *Scrt2* in the whole embryo. Modulation of *Scrt2* expression includes several other variables that are not explored in the neural tube transcriptional assays. First, ASCL1 and POU3F2 modulate expression of other transcription factors that in turn could regulate the expression of *Scrt2* (Mizuguchi et al., 2006). The resulting effect of multiple transcription factors on *Scrt2* levels would be more complex than the neural tube transcriptional assays. Furthermore, cE1 might not be the only genomic region that directs *Scrt2* expression. For example, *Scrt2* is expressed in the dorsal root ganglia, but cE1 does not drive transcription in this tissue. Thus, a yet unidentified enhancer must control *Scrt2* expression in the peripheral nervous system. Furthermore, in our 3C assay, cE1 was neither the only nor the strongest genomic element that interacted with the *Scrt2* promoter. Two other peaks also interacted with *Scrt2*. The one farther away from *Scrt2* (Ep2) was cloned and its enhancer activity was evaluated *in vivo*. Ep2 drove gene expression in the neural tube. It also presents individual target sites for ASCL1 and POU3F2. But is not conserved between birds and mammals (Supplementary Figures S2A–C). An additional site, corresponding to Ep4, also interacted strongly with the *Scrt2* promoter and is evolutionarily conserved. However, it did not present ASCL1/POU3F2 heterodimerization sites and did not drive expression in the neural tube (Supplementary Figures S2D, E).

The data presented here indicates that *Scrt2* is a direct target for ASCL1. However, the expression domain of *Scrt2* is narrower than ASCL1, which indicates that *Scrt2* mRNA levels must be regulated by additional factors in addition to ASCL1 availability.

**FIGURE 6:**
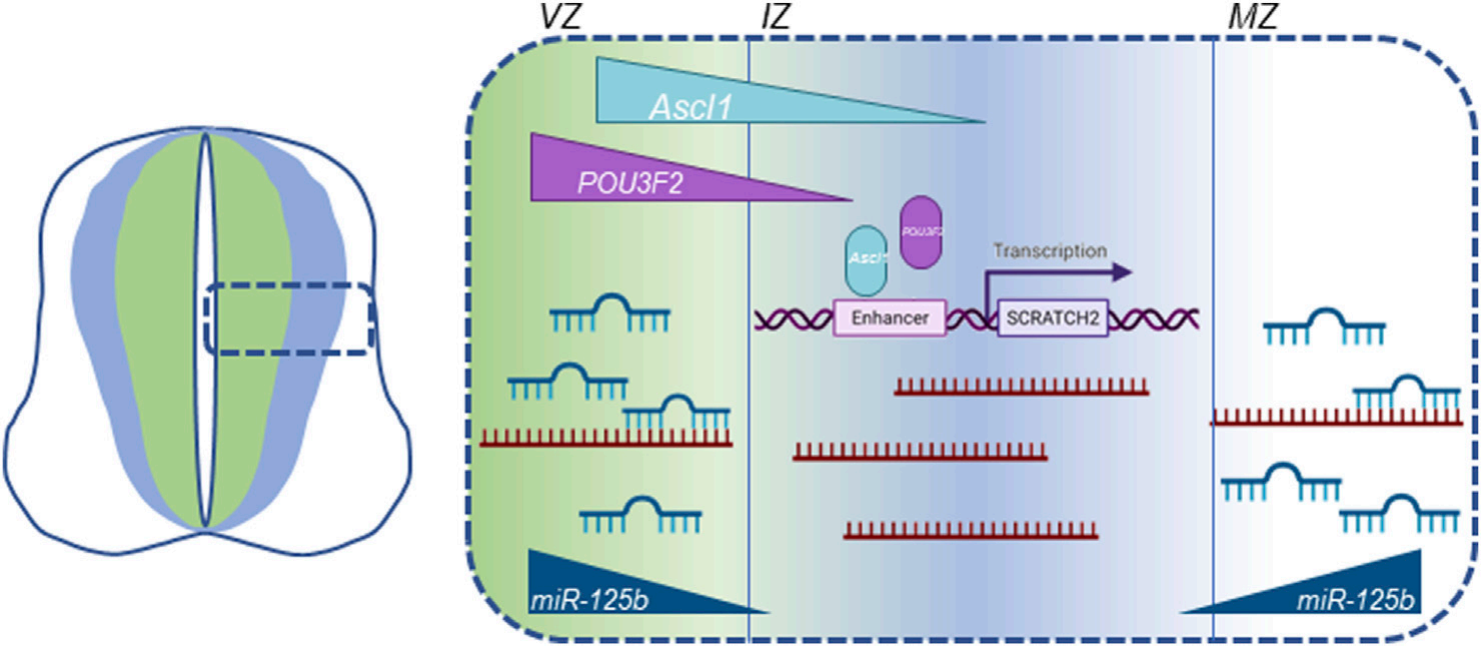
Graphic summary of all known cellular mechanisms that modulate Scrt2 transcript levels in the neural tube. *Scrt2* expression pattern is modulated by the joint action of transcription factors ASCL1, POU3F2 and miRNAs. POU3F2 (purple) and ASCL1 (light blue) are expressed more internally than *Scrt2* (dark blue) in the neural tube. ASCL1 acts through two Ascl1/POU3F2 heterodimerization motifs in cE1 enhancer to modulate *Scrt2* transcription. *Scrt2* mRNA (dark red) levels are reduced through the action of miR-125b in the ventricular and mantle zone further refining *Scrt2* expression domain (dark blue) to the intermediate zone (Goes et al., 2020). VZ, ventricular zone; IZ, intermediate zone; MZ, mantle zone.

*Scrt2* mRNA levels are directly regulated by microRNAs (Goes et al., 2020). Previous work by our group showed that the 3’ untranslated region of *Scrt2* mRNA contains target sites for miR-125b. Further, miR-125b expression in the neural tube is more external to the expression domains of *Scrt2* and *Ascl1*. Genomic removal of the target sites or reduction of miR-125b levels expanded *Scrt2* expression domain laterally. ASCL1 determines *Scrt2* transcription, while miR-125b restricts mRNA levels in the external layers of the neural tube. In this scenario, transcriptional control by proneural factors would act in concert with miRNA-mediated post-transcriptional regulation to restrict *Scrt2* expression to a narrow strip in the intermediate zone (Figure 6). This joint mechanism could also define the expression pattern of other genes relevant for neural tube development.

## Data Availability

The datasets presented in this study can be found in online repositories. The names of the repository/repositories and accession number(s) can be found in the article/Supplementary Material.

## Supplementary material

SUPPLEMENTARY FIGURE S1 Image analysis and cell quantification. **(A)** The Cellpose algorithm was used to train a model to segment the cells in ISH images of neural tubes. Then, Cellprofiler pipeline was used to segment neural tubes, identify the neural tube canal (midline) and to run Cellpose model to identify and quantify the Scrt2-positive cells. The data obtained was analysed in Python to calculate and normalize the cells distance to the midline, resulting in 2D density plots and histograms. **(B)** Overlap of the frequency distribution of *Scrt2*-positive cells in control setting (pink histogram) and after electroporation of *Ascl1*, *Pou3f2* or *Ascl1+Pou3f2* (blue histogram).

SUPPLEMENTARY FIGURE S2 Ep2 and Ep4 conservation, activity and TFBS. **(A)** Ep2 is present and conserved in other birds but is absent in mammals, fish and *Xenopus*. **(B)** The JASPAR tool found 42 ASCL1 and 1 POU3F2 binding sites in Ep2. **(C)** Ep2 can drive transcription in the neural tube of HH23 chick embryos by driving mRFP expression. (*n* = 4). **(D)** Ep4 is present and conserved across 77 vertebrates as seen by PhyloP. The most conserved region of Ep4 is absent in turkey. The JASPAR tool identified 171 sites for ASCL1 and 1 for POU3F2 in Ep4 (data not shown). **(E)** Ep4 did not drive gene reporter expression in the neural tube. mGFP was used as a positive control for electroporation efficacy. Scale bar: 50 µm.

SUPPLEMENTARY FIGURE S3 cE1 activity in different stages (HH15-HH23) of the neural tube. Embryos were electroporated in HH11-12 and collected in each indicated stage HH15 (*n* = 3), HH18 (*n* = 4), HH21 (*n* = 5) and HH23 (*n* = 5). pCDNA3.1-mGFP vector was used as electroporation effectiveness. The right side is electroporated in all images. mRFP expression under cE1 control was detected by immunolabelling. Scale bar: 50 µm.

SUPPLEMENTARY FIGURE S4 Epigenetic signatures and mE1 activity in different stages (HH15-HH23) of the neural tube. **(A)** the mouse mE1 (1600bp; blue bar) genomic region displays multiple epigenetic signatures that indicate mE1 as a transcriptionally active region in neural tissue. The ATAC-seq and histone marks for active chromatin (H3K27ac, H3K4me1, H3K9ac, H3K4me2 and H3K4me3) in neural tube of E11.5 mouse embryos are increased in mE1. In contrast, the frequency of H3K27me3 inactivity marker is not above background level. The gray highlight indicates mE1 and the position of its corresponding peaks. **(B)** Immunolabeling to detect mRFP expression in the neural tube under mE1 control. Embryos were electroporated in HH11-12 and collected in each indicated stage HH15 (*n* = 3), HH18 (*n* = 3), HH21 (*n* = 5) and HH23 (*n* = 6). pCDNA3.1-mGFP vector was used as electroporation effectiveness. The right side is electroporated in all images. Scale bar: 50 µm.

SUPPLEMENTARY FIGURE S5 Overlap of the frequency distribution of *Scrt2*-positive cells in control setting (Cas9 with scrambled sgRNAs, pink histogram) and after electroporation of Cas9 with cE1 sgRNAs (blue histogram).

SUPPLEMENTARY FIGURE S6 Luciferase assay for cE1 interaction with neural transcription factors. HEK293T cells were co-transfected with cE1-driven luciferase plasmid (pGL3-cE1) and expression vectors containing the coding sequence of *Ascl1* (*n* = 4), *Pou3f2* (*n* = 3) or both (*n* = 4). All assays were performed in three technical replicates (Unpaired Student’s *t*-test, * *p* < 0.05, ** *p* < 0.01, **** *p* < 0.0001).

SUPPLEMENTARY FIGURE S7 Dorsal view of a whole mount HH18 embryos labeled with DAPI post bilateral electroporation under V16 stereoscope. The inset shows the magnification of a representative area submitted to fluorescence intensity quantification.
