# Supplementary material for: ASCL1 promotes Scrt2 expression in the neural tube
Source: Front Cell Dev Biol. 2024 Apr 5;12:1324584. doi: 10.3389/fcell.2024.1324584 (PMC11036302; doi:10.3389/fcell.2024.1324584)

SUPPLEMENTARY 1

A

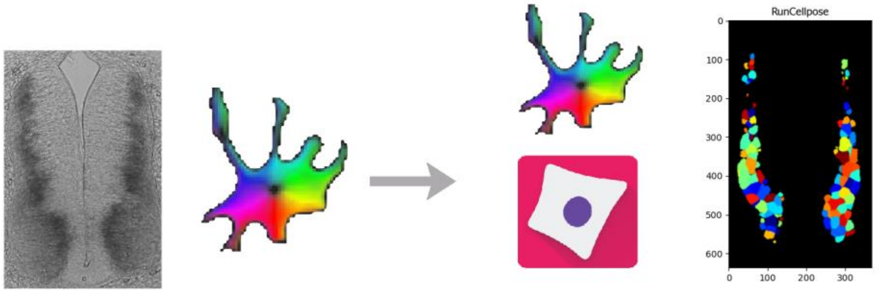

1. Train model with CellPose algorithm to segment cells in in situ hybridization images

2. Run the trained model on CellProfiler to identify and quantify Scrt2-positive cells in each hemitube.

ImageNumber	PointNumber	Distance	Angle	AreaShape_Center_Y	ObjectNumber
1	1	88.2362629758713	82.2924022520733	67.1076923076923	2
1	2	125.171548196471	75.8227510461657	74.4338461538462	1
1	3	105.086946626867	80.2454548937347	84.6128133704735	2
1	4	107.361225411436	77.5480493302071	76.5283018867925	1
1	5	88.1300678185088	82.0534487106888	82.1789883268483	2
1	6	106.730261528295	77.4360061429065	85.3181818181818	1

3. Each cell position data were normalized to the neural tube canal distance. Histograms and density plots were built. Both analyses were performed on Python.

B

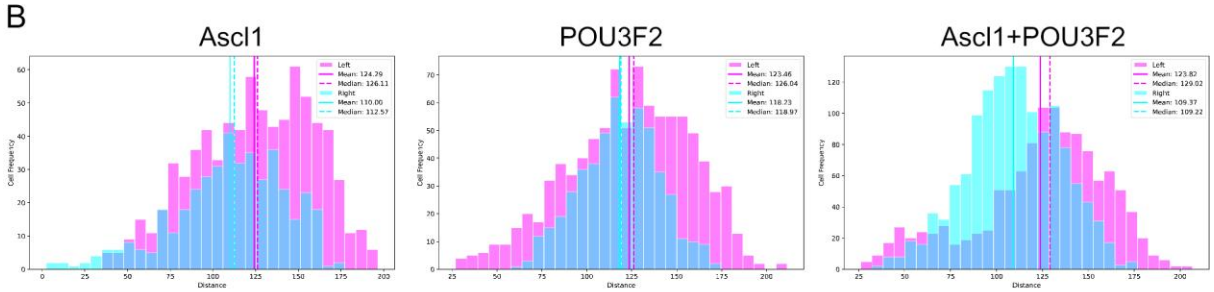

SUPPLEMENTARY 2

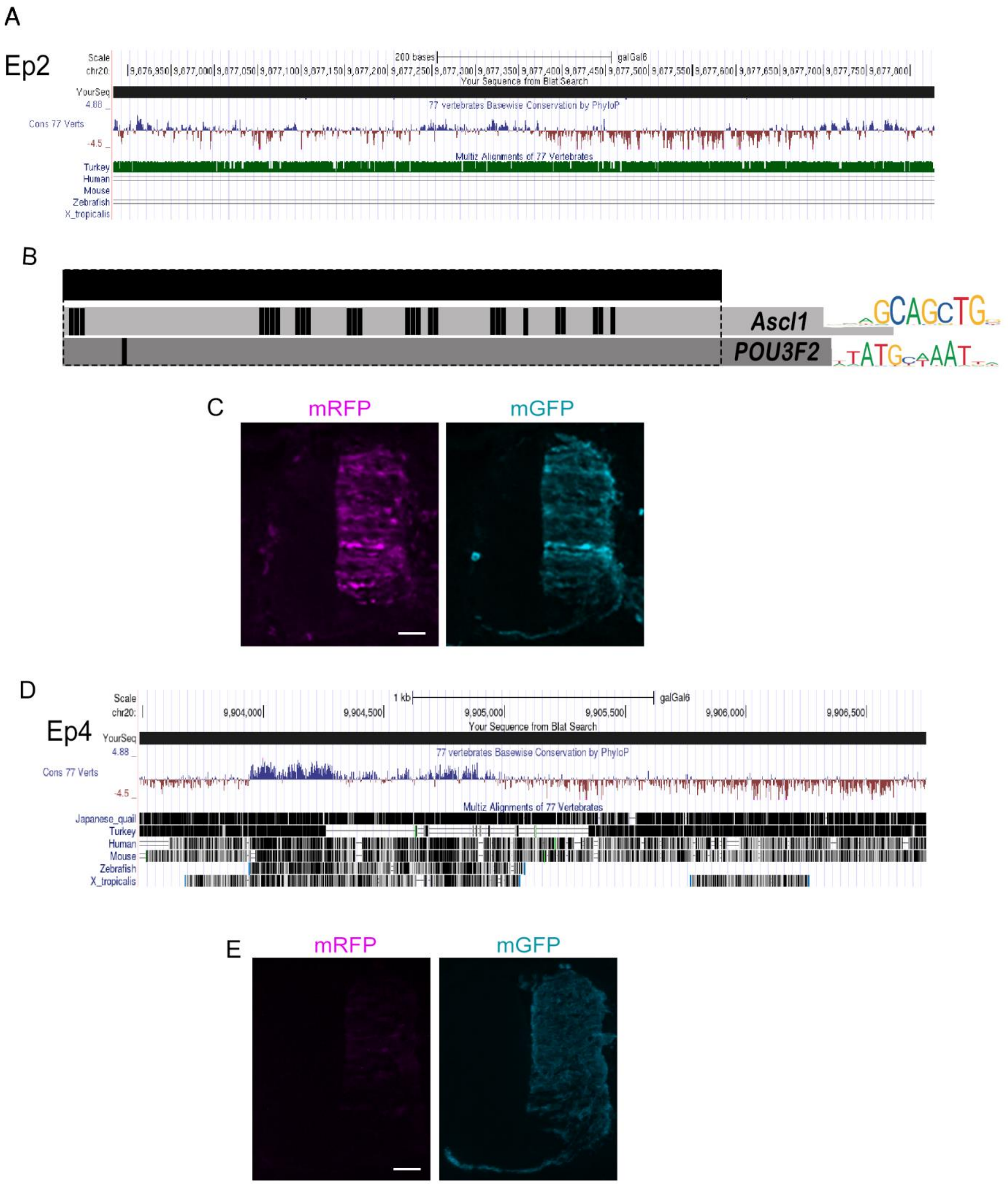

SUPPLEMENTARY 3

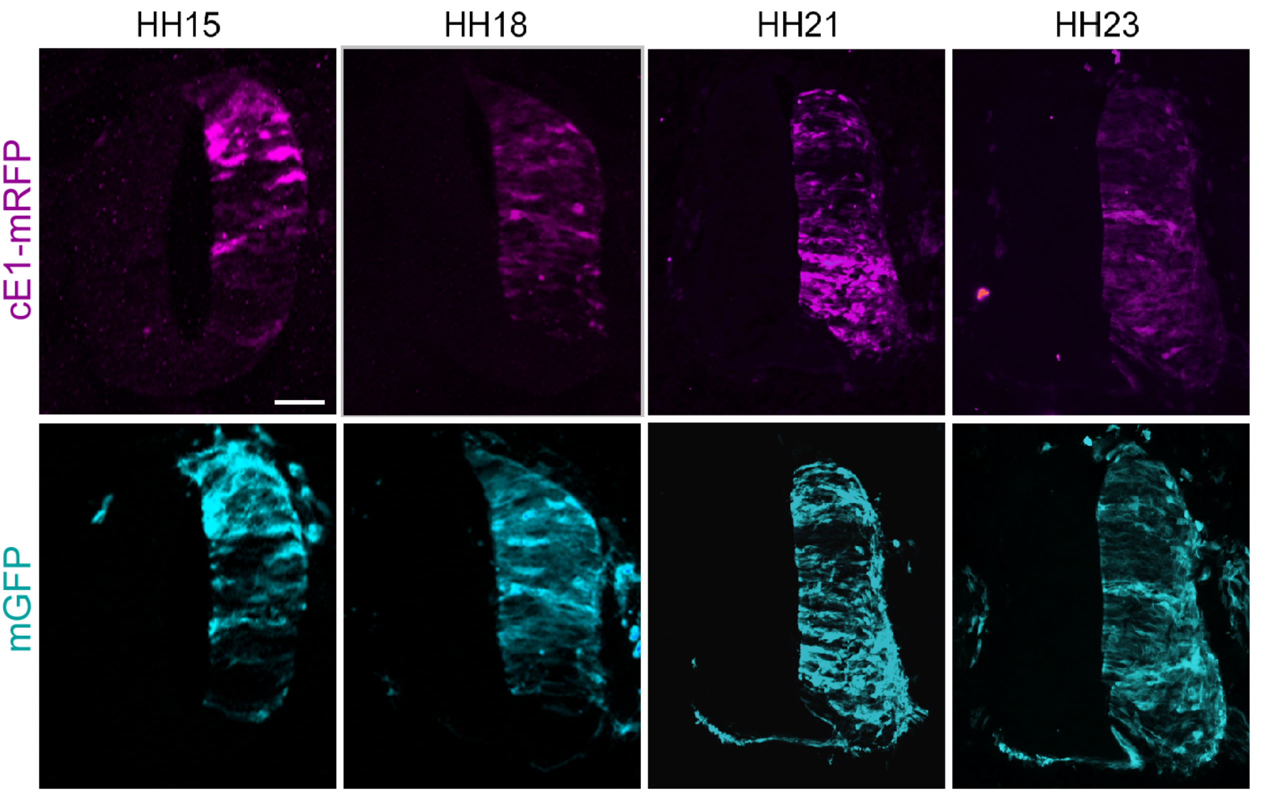

SUPPLEMENTARY 4

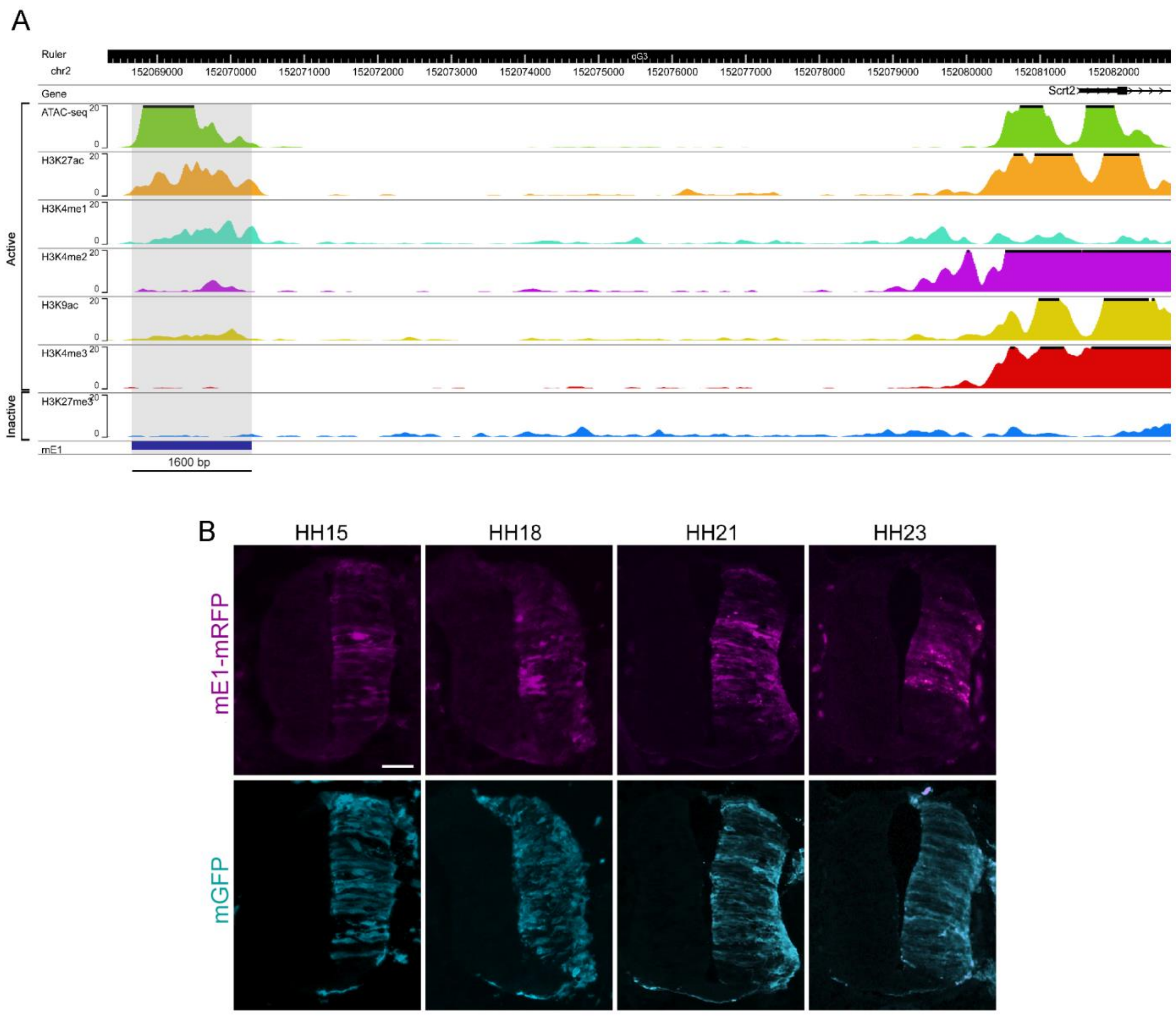

SUPPLEMENTARY 5

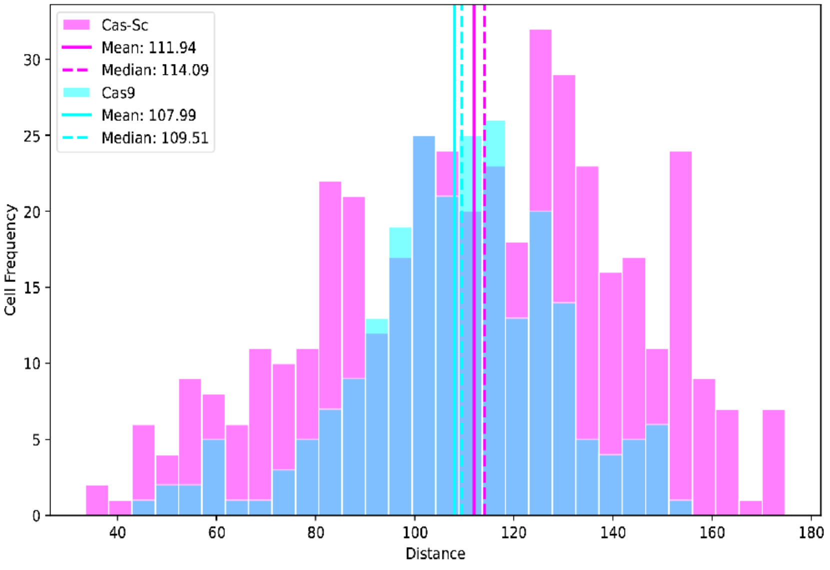

SUPPLEMENTARY 6

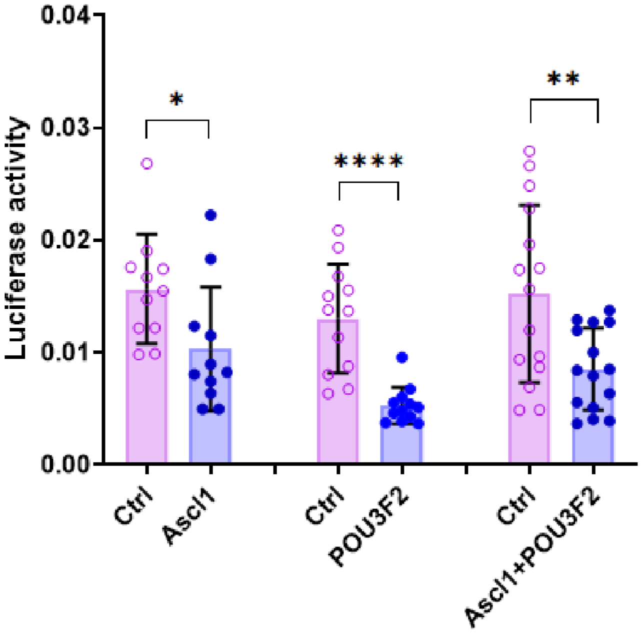

SUPPLEMENTARY 7

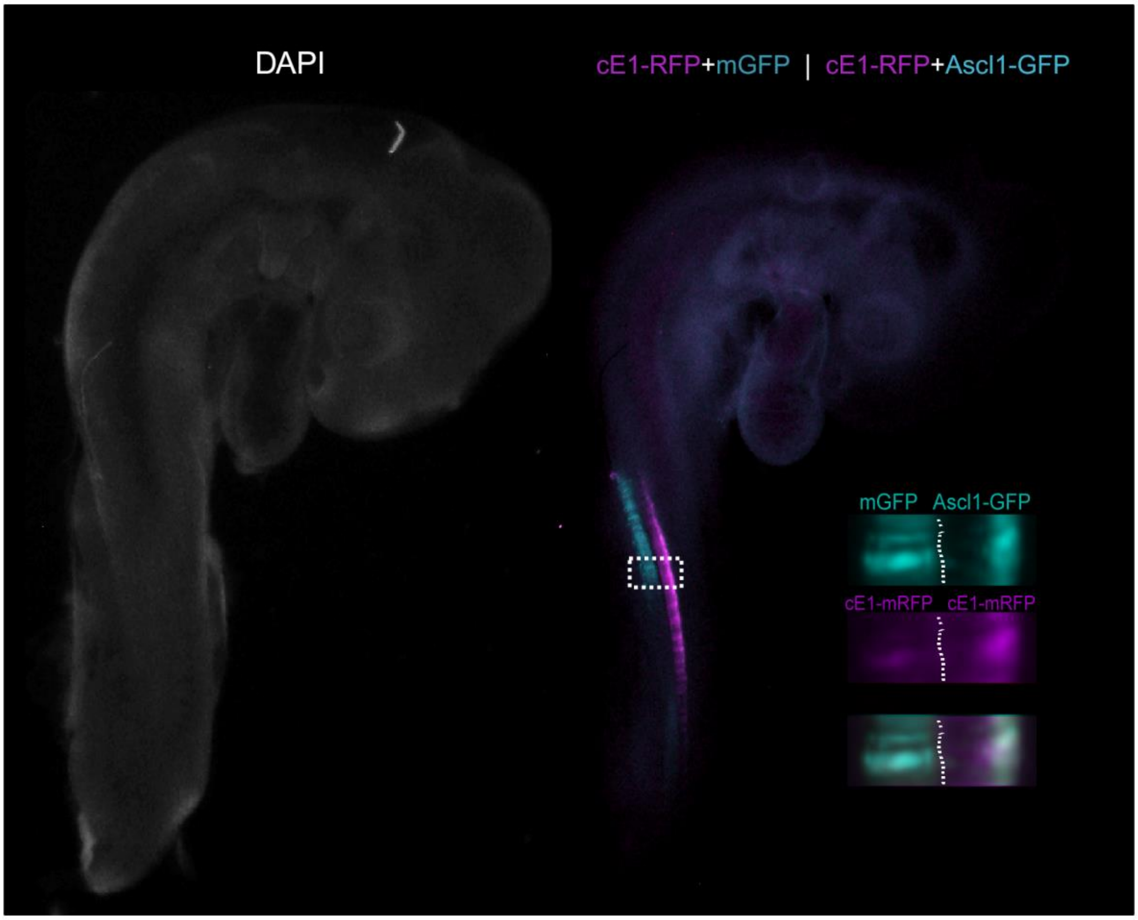

Supplement: Supplementary file 1 [file DataSheet2.pdf]
