# Supplementary material for: ASCL1 promotes Scrt2 expression in the neural tube
Source: Front Cell Dev Biol. 2024 Apr 5;12:1324584. doi: 10.3389/fcell.2024.1324584 (PMC11036302; doi:10.3389/fcell.2024.1324584)
Supplement: Supplementary file 2 [file DataSheet1.pdf]

Supplementary Material

mE1

TTTGACGCACATGTATTCATTAAGTGGATGAATGAATGAATGGGCCGTGCTCTCTGAGTCTGAACTGGA
TGTCCAAGCATTTATGTGCACTGGCTGTATACTCAGTGCTATGTACTCTTTGCTGATTTTCCAGACACTCC
TGGGAGGTCAGGGCTGCTTGGGGCTACCAAGTCCGTGACTATGCTGAGGTCACACAGTTAGCCTAGAC
CAAGCCACCACTTCTGACTGAACAGCATCCGACTCTGTAGTGTACCCCACTGACCACATTTCTCAGCCTC
CAACCCCACATGGACCCGACCTGCCTTGGCCAGACCTCCTTACTCTCCTGGTCCCATAGTCTCCTTGTAG
CTTCTCCCCCAGCCAAAGGCATCTGCCCCAACCCTCAGCCGGCACAGGCCAAGACCAAGGATGGAAGC
CATCATTTCCATACAGCTGTCCCTGGGATCTGAGCCTGGGGAGCCCCTCCATTTTCATTGAAAAGGCTG
AATTGTCTTGGCATTTTGGGGGCTGCCTGGGCGGGGCCTGGGGAGGCTATTATCCTACATTATCTCACT
CCCCTGCCCCTGAAAAGAGCCTTGACTGCCCCTCCCTCCCCTCCGCAGCCTTTTACACTTAATTACCTCAC
TCCTCACCAGCAGCCTTCAGAGGGACCACAACTGGGGCGTGAGTAATCCCGCCCCCCACACCCAGCCTG
TCTGCTAGGAGGGAATCACCCTGTGGCCTGGAAAGAAAGGAACAGACTGTGTATGTAGGCCTTCCAGC
CCGAGGACTCTGGGGCTGCTGCCACAAGGGGCAGGCGCTGACCCCAGACTCCCCGATGGCCTTTGCCA
GTCCTGGTGTCAGGCTCTCCACCTTCCTGGTCCCAGATTGTGAGCTGGGACTTTCCAGGCTGCAGAAGA
CATGTATACACGTGTGCGCGCGCGCATGCATGTGCACACACACACACACACACACACACACACACACAC
GTCTTACTGCCTTGCTTTCAAAACAGGAGGCAGGCTGAACTCCATCCCCAGAGCAGCCTGCATCACGCT
GTGCAAACATGGCTTCCAGGGTTGAAGCTCCCTCATCTTGCAGCAAATGCCTTGTGATCCTTTCTTTCCC
CTGGGCTCTACTCTTTAGTGTGCTGGAAGGGTCACTGGGCCCTGAGAGCCCTTTCTTATTCTCCAGCTGC
TAGACATAGACACTGTCTGTCAAAAGACAGGGAAAGAGTGAGGGAGGGAGGGAGTCTGAATGCATCT
TCCTTTGCTAGTGTTGGGCCCAGGTCCTACTGGGGAGCCATTGCTGGAATATAGTAACTGGGAGTGAA
GAGCCCATGGTGTAGGAATGGACCTGCTGGGAAGGTCTGCTCTGGCAAAGTGGATCCTTGGATCAGCC
TCCCCTAAGCCATCCTATCCCAGGCTGTAGGCTCCTGGCTTTGGTGCTCAGGAGCCCTGATGACCTGGA
GCATCTCAGGTCAAAGCTGCTTCTTTGCCCCCAGTGGCCCAGGTTAGGGAGACCTCCATCACTGAGAGT
CACTAATGACACTCCCATGTCACCATGGGTCACCACTGTCACCCCCGCCCCCCCAGTAAGATGGTGCAG
CTGGGGCCGAGAAGCAGCTGT

cE1

GGTACCGGCACACGGGTCCCCAAAGTCCCTCGCCAGCCCCTCTCTCCCCAGCACGGCACGGGGGAACA
ATCGCCTCCGAGCCCCGCGAGCGAGCAGGAATTATTTCCATACAGCTGCTCGGGAAGGCAGGGATGG
GGATGCAGCCATCACCCAACCGCGCTCCGGGAGAACGCCCGGGCCTTTCTTCTGGCTCTCCCCCCTCCC
CGCCGTGGTGGGGGCAGGTTTGGGGGGGCCCAGCCGCCCTGCAGTCAGCAATATTAGCACAGCTCCG
CAGGACTGATTGCAGGTTATTAACATACATTATCTCAGTCCCCTGCCTCTGAAAGGAGCCAGTTCCTATT
CCCCCTCTCCCAGCCCTCCCTCCCCCTGTCTTTTACACTTAATTACCTCCCTTATTACACCACAGCCTGGAG
AGAGAAAAAATCACAGGGCAGCGGGCAGGCAGGCAAGCTCGGCCCAACTAGGGCGTAAGTAATCGCC
CGTTCCTCGAG

M1 – The underlined indicates the mutated motifs

GGTACCGGCACACGGGTCCCCAAAGTCCCTCGCCAGCCCCTCTCTCCCCAGCACGGCACGGGGGAACA
ATCGCCTCCGAGCCCCGCGAGCGAGCAGGAATTcacacacacacacacCTCGGGAAGGCAGGGATGGGGA
TGCAGCCATCACCCAACCGCGCTCCGGGAGAACGCCCGGGCCTTTCTTCTGGCTCTCCCCCCTCCCCGCC
GTGGTGGGGGCAGGTTTGGGGGGGCCCAGCCGCCCTGCAGTCAGCAcacacacacacacacCGCAGGACT
GATTGCAGGTTATTAACATACATTATCTCAGTCCCCTGCCTCTGAAAGGAGCCAGTTCCTATTCCCCCTCT
CCCAGCCCTCCCTCCCCCTGTCTTTTACACTTAATTACCTCCCTTATTACACCACAGCCTGGAGAGAGAAA
AAATCACAGGGCAGCGGGCAGGCAGGCAAGCTCGGCCCAACTAGGGCGTAAGTAATCGCCCGTTCCT
CGAG

M2 - The underlined indicates the mutations in Ascl1 sites.

GGTACCGGCACACGGGTCCCCAAAGTCCCTcaaagagtccctaCTCCCCAGCcaaagagtccctaACAATCGCCT
CCGAGCCCCGCGAGCGAGCAGGAATaaaTTCcaaagagtccctaGGGAAGGCAGGGATGGGGATGCAGCC
ATCACCCAACCGCGCTCCGGGAGAACGCCCGGGCCTTTCTTCTGGCTCTCCCCCCTCCCCGCCGTGGTGc
aaagagtccctaGGGcaaagagtccctaTGCAGTCAGCAAgggTAgcacaggaccctaGGACTGATTGCAGGTTATT
AACATACATTATCTCcaaagagtccctaTGAAAGGcaaagagtccctaTCCCCCTCTCCCAGCCCTCCCTCCCCCT
GTCTTTTACACTTAATTACCTCCCTTATTACACCACAGCCTGGAGAGAGAAAAAATCACcaaagagtccctaG
GCAGGCAAGCTCGGCCCAACTAGGGCGTAAGTAATCGCCCGTTCCTCGAG

Table S1: Primers used for PCR amplification of control fragments spanning the 62 kb Scrt2 locus for 3C assay.

Primer	Sequence
FRAG1-F	5’ TAGAACAGGAGGGGACACAG
FRAG1-R	5’ GCTGCGTGTGAGAAATGGGG
FRAG2-F	5’ CACTGTAGGGGCTGCAAAGAG
FRAG2-R	5’ AGAGGAGGAAAGTGAAGGAGAG
FRAG3-F	5’ GTTCCAGCACCAAACTTCCC
FRAG3-R	5’ CATCCCAATGGCAGAGAAAC
FRAG4-F	5’ TAATTAAAGCGGCTCAGAACG
FRAG4-R	5’ ACGAAGGTGTAGCTGCAGTG
FRAG5-F	5’ CACTGCAGCTACACCTTCGT
FRAG5-R	5’ GTCCCCACTGCATCTCCATG
FRAG6-F	5’ GCAATAAGGCTGAAGGATTTC
FRAG6-R	5’ TTCAGCAGAAGGGACAGAAT
FRAG7-F	5’ ATTCTGTCCCTTCTGCTGAA
FRAG7-R	5’ CTTCCTCCCCTTCCATTCTC
FRAG8-F	5’ GCCTAACACAGCCTGGTATG
FRAG8-R	5’ CTTTTGGCTCACGGTCTCAT
FRAG9-F	5’ GTTTATCCCATCCCAAGAGC
FRAG9-R	5’ GAAGACGGAGCAGGAAGACT
FRAG10-F	5’ CAGCGATTCTATGCAACCCC
FRAG10-R	5’ GCATATCACCATGTCCCTCC
FRAG11-F	5’ CCTGCAGTGTGATTTACGGG
FRAG11-R	5’ CAAGGTTAAACTGCGGAGAG

Table S2: Primers used for 3C-qPCR.

Primer	Sequence
Constant-F	5’ TGACGCAGGTCAAGAGTCAA
Peak1-F	5’ TGGATCCCGCAAACCAATG
Peak2-F	5’ GGGCTGGCTCTGTCTGTG
Peak3-F	5’ CTGGCAGAGAGCAGGATGT
Peak4-F	5’ GGCTTCAGCTGGAAAAACAG
Peak5-F	5’ ACAGGTGACCTTGGACTTGG
Peak6-F	5’ ATCCTCATCCCTCCCTGTG

Table S3: Primers used to clone the fragments used in this work.

Name	Sequence
mE1-F	5’ GGTACCCACATGTATTCATTAAGTGGATGA
mE1-R	5’ CTCGAGCAGCTGCTTCTCGGCCCCAGCTGCAC
cE1-F	5’ GGTACCGGCACACGGGTCCCCAAAGTC
cE1-R	5’ CTCGAGGAACGGGCGATTACTTACGCC
USE-F	5’ TCTTACGCGTGCTAGCCCCTCAGCACCCTTGGGAGC
USE-R	5’ ATCCGAGATCTCGAGCCCACTCCCGTTCCCAGCCTG
USE1-F	5’ TCTTACGCGTGCTAGCCCGACCTGCTGCTTTCCACCTC
USE1-R	5’ ATCCGAGATCTCGAGCCCTGGGACGGGCAAGGAGCA
USE3-F	5’ TCTTACGCGTGCTAGCCCTGCAAACGTGCCTTCAAGA
USE3-R	5’ ATCCGAGATCTCGAGCCCACTCCCGTTCCCAGCCTGGGGC
Ep2-F	5’ TCTTACGCGTGCTAGCCCAGGAGCGCATCATGCAGGCACT
Ep2-R	5’ ATCCGAGATCTCGAGCCCGCTCACAGCCCTGCTTCCCCTC
Ep4-F	5’ GTACCGAGCTCTTACGCGTGCACCCAAACCCTCCAGGAC
Ep4-R	5’ ATCTCGAGCCCGGGTCGACGCACCAAGACACCCTGGGAC

Table S4: oligos used as guides in CRISPR/Cas9 or dCas9 assays.

Name	Sequence
cE1 sgRNA1-F	5’ AGTCGCCTGCCCGTCCCAGGCACAC
cE1 sgRNA1-R	5’ AAACGTGTGCCTGGGACGGGCAGGC
cE1 sgRNA2-F	5’ AGTCGGCCGTGGTGGGGGCAGGTTT
cE1 sgRNA2-R	5’ AAACAAACCTGCCCCCACCACGGCC
cE1 sgRNA3-F	5’ AGTCGAGGCAAGCTCGGCCCAACTA
cE1 sgRNA3-R	5’ AAACTAGTTGGGCCGAGCTTGCCTC
cE1 sgRNA1 Scrambled-F	5’ AGTCGGCCAACGCCTCGTCCGCACC
cE1 sgRNA1 Scrambled-R	5’ AAACGGTGCGGACGAGGCGTTGGCC
cE1 sgRNA2 Scrambled-F	5’ AGTCGGGTTGTGGTGCGCGGTAGGC
cE1 sgRNA2 Scrambled-R	5’ AAACGCCTACCGCGCACCACAACCC
cE1 sgRNA3 Scrambled-F	5’ AGTCGGCAAGCCGAACCTAACGTGC
cE1 sgRNA3 Scrambled-R	5’ AAACGCACGTTAGGTTCGGCTTGCC

Data availability

Data	Repository	Accession	Reference
H3K27ac and IgG of HH23 neural tubes	Github	https://github.com/goescp/Scrt2data	Current work
Epigenetic profile of E11.5 mouse neural tube	ENCODE Project https://www.encodeproject.org/	ATAC-seq ENCSR215ZYV; H3K4me3 ENCSR215ZYV; H3K27ac ENCSR215ZYV; H3K4me1 ENCSR215ZYV; H3K9ac ENCSR215ZYV; H3K4me2 ENCSR215ZYV; H3K27me3 ENCSR215ZYV	Luo et al., 2020
